# The Choice of Bromides in the Treatment of Epilepsy

**Published:** 1899-08-19

**Authors:** 


### THE CHOICE OF BROMIDES IN THE TREATMENT OF EPILEPSY.

A series of experiments have lately been carried out at Hanwell by Dr. J. G. Smith,[1] assistant medical officer, London County Asylum, to determine the utility of bromide of strontium, as compared with the corresponding potassium salt, in the treatment of epilepsy. The plan followed in each instance was to begin with 15 grains three times daily, and to increase this amount, at intervals of one week, if necessary to 20 grains, 30 grains, and even 40 grains three times daily, until the seizures were under control. It was only necessary in one instance to go beyond 40 grains, and in that case the patient became stuperose and unsteady in gait under the larger dose, while no abatement took place in the number of the seizures, and in this case the same result followed the use of each salt. On the whole there was only a very slight difference between the effects produced by the two drugs, though what difference there was was clearly in favour of the strontium as regards the absolute number of fits. It was also found that the seizures of the patients when under this drug were in some instances of a milder type than when they were under the potassium salt, and that the tendency to the appearance of a bromide rash was much less marked in the case of the strontium than the potassium bromide. On the other hand a larger quantity of the strontium was required than of the potassium to get the fits under control. Summing the matter up, then, it would seem that whilst bromide of strontium is in some cases of greater value than bromide of potassium, yet the latter, on account of its more rapid action, its more durable effects, and lastly, its cheapness, is to be regarded as the more generally useful drug in the treatment of epilepsy. We would add, however, that even if bromide of strontium did not show any marked superiority it might still be useful as a change from the weary monotony in physic which some epileptics have had to put up with; but in view of the statement made by Dr. Smith, that in some cases the strontium salt did seem to be the best, not merely in diminishing the frequency, but in modifying the character of the attacks, and especially in view of what he says as to its being less likely to cause bromide rash, we think that it will be a common sense plan, whenever a case of epilepsy, from some cause which is not obvious and remediable, does not make good progress under potassium, to try strontium. There are some things that nothing short of actual experiment will decide, and among them is the question, Which salt will best suit a given case of epilepsy?

[1] Lancet, Aug. 12.

